# Psychosocial and Environmental Correlates of Sedentary Behaviors in Spanish Children

**DOI:** 10.1155/2017/4728924

**Published:** 2017-04-27

**Authors:** S. Aznar, M. T. Lara, A. Queralt, J. Molina-Garcia

**Affiliations:** ^1^PAFS Research Group, Faculty of Sports Sciences, University of Castilla-La Mancha, Toledo, Spain; ^2^Department of Nursing, University of Valencia, Valencia, Spain; ^3^Department of Teaching of Musical, Visual and Corporal Expression, University of Valencia, Valencia, Spain

## Abstract

*Purpose*. To evaluate children's psychosocial and environmental factors associated with sedentary behavior (SB).* Method*. The study involved a total of 420 children (mean 9.2 years; 52.9% girls) from the community of Madrid, Spain. SB and physical activity (PA) were objectively measured using accelerometers. TV viewing and potential correlates were assessed by questionnaire. Mixed-model regression analysis, adjusted for clustering within school locations, evaluated the relation of each independent variable with SBs.* Results*. Girls showed higher levels of SB than boys, whereas boys reported more TV viewing (*p* < .001 in all cases). Regression analysis showed that MVPA levels were negatively related to objective SB measurement in both boys and girls (*p* < .001). Parent and friend support to PA were negatively associated with SB on weekdays in boys and girls, respectively (*p* < .05). In the boys' group, parental professional level was a positive predictor of SB on weekend days (*p* = .011). Boys with more positive neighborhood perceptions spent less time watching TV (*p* < .001), whereas mother's leisure-time PA level was a negative correlate of TV viewing in girls' group (*p* < .01).* Conclusion*. Different psychosocial and environmental correlates of SB were identified. Present findings are promising targets for interventions to improve children's health.

## 1. Introduction

Sedentary behaviors refer to any waking behavior characterized by an energy expenditure ≤1.5 MET (i.e., sitting or reclining posture) [1]. Current literature has shown that lack of physical activity (PA) and high sedentary behavior (SB) are independently related to a greater prevalence of noncommunicable diseases and mortality in children [2, 3]. According to this evidence, these two behaviors should be analyzed separately in childhood [4]. In recent years, there has been a great concern about the excessive amount of time children spent in SB. Children and youth who are sedentary have greater fat mass, higher body mass index, and greater risk of becoming obese, regardless of how much physical activity they perform when they are not sedentary [5–7].

Children's sedentary behavior can consist of leisure-time activities (e.g., TV viewing, Internet, and playing computer games) or sitting during school time. In recent years, it has been stated that too much prolonged sitting time may be most harmful than total sedentary time [8] and children are more likely to interrupt their SB much more regularly. Therefore, it seems that to interrupt children's SB could be a good intervention strategy.

In children, most of the SB available data is based on self-reported measures such as questionnaires and diaries. This situation is a limitation because self-reported SB grossly underestimates total sedentary time [9]. Children do not usually have structured and habitual activity patterns [10, 11] and nowadays the habit of “media multitasking” is very popular among youth. Thus, accelerometers are one of the objective instruments to accurately assess PA and sedentary behaviors [12]. Using combination of self-report with objective assessment is a mean to better understand the context where such behavior occurs.

In a current study with accelerometry [13], high proportions of children from different European countries did not meet moderate-to-vigorous physical activity (MVPA) recommendations of at least 60 minutes/day and showed high levels of sedentary time. Furthermore, girls showed lower levels of PA and spent more time being sedentary than boys. Apart from gender differences, literature indicates that PA and sedentary behaviors differ between weekdays and weekend days [14]. However, accelerometers do not provide information about the type of SB people engage in nor of the social context where sedentary activities take place; therefore a combination of self-report and accelerometers are encouraged.

In these days, developing successful interventions to increase PA and reduce sedentary time is one of the major research priorities for children [15]. Nevertheless, few studies have analyzed correlates of objectively measured PA and sedentary time [16], particularly differentiating between girls and boys, considering weekdays versus weekend days and aimed at identifying the determinants of SB with an ecological model perspective.

Current research on the correlates of SB has focused on the differentiation between personal, psychosocial, and environmental factors through multilevel ecological models [17]. In order to provide an empirical basis for effective policies to increase PA and reduce SB in children, it is crucial to understand the effects of the psychosocial and environmental factors on these behaviors [15].

Based on an ecological perspective that behaviors are influenced by factors at multiple levels [18], the present study evaluated personal, psychosocial, and environmental correlates of SB, in a sample of Spanish children using accelerometers and self-reported measures of SB.

## 2. Methods

### 2.1. Participants and Recruitment

The study involved a total of 420 children (222 girls, 198 boys) aged 8–10 y, from Spain part of the European Youth Heart Study (EYHS) [19]. The EYHS is a school-based, cross-sectional study designed to examine the interactions between personal, environmental, and lifestyle influences on the risk factor for future cardiovascular diseases in several European countries.

All eligible schools were stratified according to location (urban, suburban, and rural) and the socioeconomic profile of the uptake area (high, middle, and low). The study sample was randomly selected using a two-stage cluster sample procedure, with schools in Madrid as primary sampling units. The secondary units were the children within the schools, and equal numbers of children were sampled randomly from each school. Prior to data collection, children's verbal consent was obtained to participate in this study.

Ethical approval was obtained by the Health Institute Carlos III in Madrid, Spain, and parental informed consent was obtained for each participant prior to data collection.

## 3. Measures

### 3.1. SB and PA Measurement

PA was measured during 6 consecutive days using the MTI accelerometer, model GT1 M activity monitor. All children wore the accelerometer during the daytime, except while bathing or during other aquatic activities. Verbal and written instructions for care and placement of the monitor were given to both, children and their parents. Validation studies examining this accelerometer suggest that it is a valid and reliable measurement of children's PA [20–22]. For data to be considered valid two criteria were established: a minimum of data for a period of 4 days including one weekend day and a minimum of 10 registered hours of data per day [23]. Count ranges for the various activity intensities were as follows: 0 to 499 for sedentary, 500 to 1999 for light, 2000 to 2999 for moderate, 3000 to 4499 for vigorous, and 4500–32767 for very vigorous activities according to Andersen et al. (2006) [24]. To analyze the accelerometer data, Kinesoft software, developed specifically for the Actical and ActiGraph accelerometers, was used. The outcome variables were expressed as average intensity (counts/minute) and amount of time (minutes/day) spent at different PA-intensity categories. We calculated mean counts per minute by dividing the sum of total counts per epoch (15 seconds) for a valid day by the number of minutes of wear time in that day across all valid days. We excluded from the analysis bouts of 20 continuous minutes of activity with intensity counts of 0, considering these periods to be nonwearing time [25].

TV viewing, as in previous EYHS analyses [26], was measured asking children the number of hours per day spent watching TV after school: none (0), <1 (1), 1-2 (2), 2-3 (3), and >3 (4).

### 3.2. Potential Correlates of SB

#### 3.2.1. Parents' Physical Activity

Both parents were asked about how many days, in a typical week, they spent at least 30 minutes in leisure-time PA. Possible responses were as follows: 1 (“No days a week”), 2 (“1-2 days a week”), 3 (“3-4 days a week”), 4 (“5-6 days a week”), and (5 “Every day”).

#### 3.2.2. Parental Professional Level

Parents were asked about their current occupation. According to previous studies [27], three professional levels were derived: unskilled worker/unemployed (1 = low), skilled worker (2 = medium), and managerial (3 = high) level. The highest professional level of parents was used.

#### 3.2.3. Perceived Personal, Psychosocial, and Environmental Measures

The validated questionnaire from EYHS [28] was used to assess the following constructs.

*Enjoyment and Physical Competence Perception*. This measure is based on eight items, using a response format from 1 (“definitely no”) to 3 (“definitely yes”). Example items are as follows: “I usually prefer to watch rather than play games” or “I feel that I am better than most other kids my age at games and sports.” 

*Physical Activity Social Support*. Two three-item scales were used to analyze parent and peer support. Example items are as follows: “How often does your mum or dad take you to exercise or play sports?” and “How often do your friends ask you to play out with them?” Items were rated from 1 (“hardly ever or never”) to 4 (“every day”). 

*Neighborhood Environment Perceptions*. Environmental influences were assessed through six items. Item examples are as follows: “It is safe to walk or play alone in my neighborhood during the day” and “There is somewhere at home I can go out and play.” Response options were from 1 (“definitely no”) to 3 (“definitely yes”).

## 4. Statistical Analyses

Data analyses were carried out using SPSS 22.0, and the level of significance was set at *p* < .05. Descriptive analyses were performed for the study variables. *t*-test was used to examine gender differences. Mixed effects regression models (using SPSS MIXED) evaluated the relation of each independent variable with SBs, adjusting for all covariates, as fixed effects and participant clustering in school locations (per recruitment procedures) as a random effect. Gender variable and week period (weekdays and weekend days) were considered in the analysis. Significance levels and *t* statistics from the adjusted mixed models were presented instead of *β* estimates and confidence intervals in order to provide common indicators for comparing relative magnitudes of association across variables.

## 5. Results

Table 1 shows descriptive statistics by gender for the variables analyzed in the present study. Girls showed higher levels of SB than boys on weekdays and weekend days (*p* < .001). However, boys reported more TV viewing (*p* < .001) and showed higher levels of MVPA on weekdays and weekend days (*p* < .001).

### 5.1. Correlates of SB

Mixed-model regressions showed there were significant associations with several independent variables and SBs (see Table 2). MVPA levels were negatively related to objective SB measurement on weekdays and weekend days, in both boys and girls (*p* < .001). Parent and friend support to PA were negatively associated with SB on weekdays in boys and girls, respectively (*p* < .05).

Furthermore, in the boys' group, parental professional level was a positive predictor of SB on weekend days (*p* = .011). This relationship was not found for girls. According to regression analysis, boys with more positive neighborhood perceptions spent less time watching TV (*p* < .001).

Regarding girls, regression analysis showed that mother's leisure-time PA level was a negative correlate of TV viewing (*p* < .01). Enjoyment and physical competence perception were nonsignificant predictors in boys and girls.

## 6. Discussion

Despite the scientific evidence demonstrating that high SB and low PA are two independent behaviors related to a greater risk of disease and mortality in children, our study has shown that both behaviors are significantly negatively correlated in children. In our study, MVPA levels were negatively related to objective SB measurement on weekdays and weekend days in both boys and girls. These results suggest future intervention studies should include together these two behaviors in order to achieve better health outcomes among children. For all children, perceived parental and friends' support to PA on weekdays were negatively associated with SB (i.e., the more support they perceived the less SB they had and vice-versa).

Parental support for children's PA may take many forms, including parental modelling, attitudes, or direct practical help, and has been found to be important in influencing PA in young people in numerous published studies [29, 30]. Unquestionably the environment of a young person, especially a small child, is strongly influenced by their parents' choices. If the immediate family environment does not provide PA opportunities, parents can play an important role in providing a solution (i.e., transport, money, equipment, etc.).

Parental support is often being studied as a sum of the mother's (or female guardian) and father's (or male guardian) influence on a child's physical behavior. However, some studies have focused on the specific gender effect, that is, the impact of maternal and paternal modelling on PA levels in boys and girls, respectively. Schoeppe et al. (2016) [31] reported a specific gender effect, in which mothers' and fathers' sports participation were significantly associated with girls' and boys' leisure-time PA, respectively. However, there is paucity of research on the influence of parental support on children's SB. Our study found that TV watching time was significantly negatively associated with maternal support for PA in girls. Therefore, emphasis can be placed on educating parents about the importance of PA for both, themselves and their children, and informing mothers about the important role they have in reducing their daughters' SB. Moreover, it seems very convenient also to provide parents, and particularly mothers, with possibilities and alternatives for all to be physically active in the neighborhood or nearby community.

Support of friends has also been identified by others as a significant positive influence on PA in young people, particularly adolescents [32, 33]. Peer influence is potentially a key factor in shaping the attitudes and behaviors of young people, especially adolescent girls, towards PA [33, 34]. Our study contributed to the literature a more concrete influence, where peers' influence on PA was a significant negative influence on children's SB. Therefore, it seems that efforts could be directed at addressing the barriers that exist towards PA within young people's social circles and these should be gender-specific. In a longer term approach, if children of either gender have positive experiences with exercise and PA from an early age and see it as a pleasurable and worthwhile pursuit, they will be more likely to remain active into adolescence and in turn become positive role models for their peers. This underlines the importance of priming children with positive attitudes towards PA at an early age. Therefore, both short- and long-term approaches will need to be taken to address the issue of peer support and modelling.

Moreover, a perception of a safe environment seems important for children's PA, particularly boys, to reduce their TV watching time. This seems logical, when playing outside is required. The influence of environmental features on PA behavior has attracted great attention in recent years among adults [35]. Nevertheless, there are still few studies that have examined the relationship between built and social neighborhood environments and PA levels, especially in children. The present findings are consistent with those of a recent study showing that children's perceived autonomy and family environment are positively associated with leisure-time PA [36]. More research is needed to further deepen the relationship between environmental characteristics and PA and SB behaviors in young people.

To summarize, our study found important socioenvironmental determinants of SB with an ecological model perspective. Consideration of these socioenvironmental influences on children's SB may be an important addition to current guidelines. Ideally, such guidelines should be part of a multidimensional strategy for promotion of a supportive environment for PA within families, schools, and communities. Particular attention must be placed on reducing girls' SB.

## Figures and Tables

**Table 1:** Descriptions of sedentary behavior, moderate-to-vigorous physical activity, and correlate variables by gender. Study is conducted in Madrid, Spain, in 2008–2010.

	Range	All (*n* = 420)		Boys (*n* = 198)		Girls (*n* = 222)		*p* ^a^
		*M*	SD	*M*	SD	*M*	SD	
*SB*								
SB (min/day)	810–1255	1045.81	76.51	1016.16	72.59	1072.26	70.07	**<.001**
Weekday SB (min/day)	819–1245	1049.40	78.32	1018.98	73.47	1076.53	72.45	**<.001**
Weekend SB (min/day)	726–1315	1043.49	104.26	1016.90	107.70	1067.21	95.24	**<.001**
TV viewing	0–4	1.15	.90	1.33	.97	.98	.80	**<.001**
*MVPA*								
MVPA (min/day)	30–320	140.22	48.76	162.05	50.01	120.75	38.33	**<.001**
Weekday MVPA (min/day)	34–326	142.93	50.70	164.45	52.41	123.73	40.48	**<.001**
Weekend MVPA (min/day)	14–375	133.06	63.37	153.89	68.60	114.48	51.79	**<.001**
*Correlate variables*								
Enjoyment-competence	1–3	2.48	.30	2.54	.28	2.43	.31	**<.001**
Mother's physical activity	1–5	2.49	1.47	2.45	1.35	2.53	1.56	.588
Father's physical activity	1–5	2.44	1.39	2.29	1.33	2.58	1.43	**.045**
Parental professional level	1–3	2.09	.74	2.06	.76	2.11	.72	.488
Parent support	1–4	1.93	.67	2.03	.70	1.84	.63	**.004**
Friend support	1–4	2.16	.71	2.32	.75	2.02	.65	**<.001**
Neighborhood environment perceptions	1–3	2.31	.30	2.28	.30	2.34	.31	**.036**

*Note*. SB = sedentary behavior; MVPA: moderate-to-vigorous physical activity. ^a^For gender.

**Table 2:** Mixed-model regression results for relationship between independent variables and sedentary behaviors for boys and girls. Study is conducted in Madrid, Spain, in 2008–2010.

	Boys								Girls							
	SB		Weekday SB		Weekend SB		TV viewing		SB		Weekday SB		Weekend SB		TV viewing	
	(min/day)		(min/day)		(min/day)				(min/day)		(min/day)		(min/day)			
	*t*	*p*	*t*	*p*	*t*	*p*	*t*	*p*	*t*	*p*	*t*	*p*	*t*	*p*	*t*	*p*
MVPA (min/day)	−17.63	**<.001**	**—**	**—**	**—**	**—**	**—**	**—**	−21.43	**<.001**	**—**	**—**	**—**	**—**	**—**	**—**
Weekday MVPA (min/day)	**—**	**—**	−18.42	**<.001**	**—**	**—**	−.51	.613	**—**	**—**	−19.83	**<.001**	**—**	**—**	.05	.961
Weekend MVPA (min/day)	**—**	**—**	**—**	**—**	−19.88	**<.001**	**—**	**—**	**—**	**—**	**—**	**—**	−23.76	**<.001**	**—**	**—**
Enjoyment-competence	.59	.558	.35	.730	.62	.539	−.28	.780	1.09	.277	1.05	.295	1.00	.320	−.64	.525
Mother's physical activity	−.64	.524	−.54	.593	−.36	.722	−.24	.808	−.19	.851	−.25	.801	−.30	.762	−2.62	**.009**
Father's physical activity	−.15	.882	.20	.843	−.75	.455	−.49	.623	.68	.499	1.14	.256	.07	.945	.36	.717
Parental professional level	2.37	**.019**	1.16	.247	2.57	**.011**	.35	.724	1.09	.277	.59	.557	1.86	.064	−1.75	.082
Parent support	.58	.563	1.60	.111	−.44	.659	.77	.441	−2.37	**.019**	−2.65	**.009**	−1.26	.210	−1.41	.161
Friend support	−1.91	**.050**	−2.14	**.034**	−.83	.408	.93	.354	.05	.958	.48	.633	−.92	.357	.64	.525
Neighborhood environment perceptions	.85	.398	.61	.540	1.18	.241	−4.77	**<.001**	−1.48	.141	−1.09	.279	−1.73	.085	−.24	.812

*Note*. SB = sedentary behavior; MVPA: moderate-to-vigorous physical activity.
